# Classifying disaster risk reduction strategies: conceptualizing and testing a novel integrated approach

**DOI:** 10.1186/s12992-023-01006-8

**Published:** 2024-01-08

**Authors:** Mariya Dimitrova, Megan Snair

**Affiliations:** 1 University of Gothenburg, Gothenburg, Sweden; 2 SGNL Solutions, Evergreen, Colorado, United States

**Keywords:** Disaster risk reduction, DRR, Categorization, Comprehensive, Framework

## Abstract

**Background:**

Although disaster risk reduction (DRR) addresses underlying causes and has been shown to be more cost-effective than other emergency management efforts, there is lack of systematized DRR categorization, leading to insufficient coherence in the terminology, planning, and implementation of DRR. The aim of this study was to conceptualize and test a novel integrated DRR framework that highlights the intersection between two existing classification systems.

**Methods:**

Grounded theory was used to conceptualize a novel DRR framework. Next, deductive conceptual content analysis was used to categorize interventions from the 2019 Cities100 Report into the proposed DRR framework. The term “connection” indicates that an intervention can be categorized into a particular section of the novel integrated approach. A “connection” was determined to be present when the intervention description stated an explicit connection to health and to the concept within one of the categories from the novel approach. Further descriptive statistics were used to give insight into the distribution of DRR interventions across categories and into the application of the proposed framework.

**Results:**

The resulting framework contains nine intersecting categories: “hazard, prospective”, “hazard, corrective”, “hazard, compensatory”, “exposure, prospective”, “exposure, corrective”, “exposure, compensatory”, “vulnerability, prospective”, “vulnerability, corrective”, and “vulnerability, compensatory”. The thematic analysis elucidated trends and gaps in the types of interventions used within the 2019 Cities100 Report. For instance, exposure-prospective, exposure-compensatory, and vulnerability-compensatory were the most under-utilized strategies, accounting for only 3% of the total interventions. Further descriptive statistics showed that upper middle-income countries favored “hazard, corrective” strategies over other DRR categories while lower middle-income countries favored “exposure, corrective” over other DRR strategies. Finally, European cities had the highest percentage of DRR connections (51.39%) compared to the maximum possible DRR connections, while African cities had the lowest percentage of DRR connections (22.22%).

**Conclusions:**

The study suggests that the proposed DRR framework could potentially be used to systematically evaluate DRR interventions for missing elements, aiding in the design of more equitable and comprehensive DRR strategies.

## Background

Disasters are hazards that significantly disturb the functioning of a community, region, nation, or society, causing human, material, economic, or environmental losses and effects [1]. The severity or impact depends on the intersection of the hazard with conditions of exposure, vulnerability, and the capacity of a community. As climate change intensifies, disasters are also expected to increase in frequency and severity [2].

Disaster Risk Reduction (DRR) as a practice differs from traditional disaster management efforts in that DRR not only considers disasters in terms of preparing for or responding to them, but also anticipates their future effects, attempting to reduce the associated risk [3]. DRR aims to be preventative and holistic by addressing underlying drivers, such as vulnerability, and has been shown to be more cost-effective than other emergency management efforts [3–5]. DRR is also interwoven with public health risks, especially related to infectious disease outbreaks, as linkages between fragile states, natural, or manmade disasters and the emergence of pathogens are well established [6]. Going further upstream in tackling these issues can be beneficial in reducing the impacts these public health risks may bring. The 2030 Agenda for Sustainable Development addresses the need for pre-disaster prevention and planning in Sustainable Development Goal Number 3, and Target 3.d as a means of implementation by calling for increased “early warning, risk reduction and management of national and global health risks” to strengthen the capacity of all countries [7]. Similarly, in paragraph 17, the United Nations (UN) Sendai Framework for DRR calls to:

*“prevent new and reduce existing disaster risk through the implementation of integrated and inclusive … measures that prevent and reduce hazard exposure and vulnerability to disaster, increase preparedness for response and recovery, and thus strengthen resilience”* [8].

However, compared to disaster management, less progress has been made in DRR whether in preventing risk, addressing underlying drivers of disasters [9], or strengthening resilience to risk [10]. This lack of progress is multifaceted, but includes poor governance and political barriers as some of the key challenges preventing better implementation of these types of DRR frameworks. The incentives within our political systems often conspire against prevention and preparedness, and certain political dynamics may push towards or away from preparedness actions [11]. Simply investing in preparedness frameworks and actions has been slow due to a lack of political will in countries from Kenya [12] to the United States [13] to Pakistan [14], with most governments allocating investments to disaster response efforts because those are more visible and leaders often think will have more value to constituents. Poor governance, environmental degradation, poverty, and climate change can also compound one another to make a country’s disaster management more focused on response rather than preparedness and prevention [15].

Prioritizing action further upstream in DRR activities can be even more politically conflicting than traditional preparedness efforts. Because the benefits may take years to realize, investment in DRR is often seen as financially irresponsible and less of an immediate priority, especially for policymakers [10, 16, 17]. Globally, DRR has been underutilized, such as in the United States [18], India [19], Australia [20], and Pakistan [21]. In addition to political barriers and the longer timeline to realize benefits, other barriers to implementing DRR include lack of DRR expertise or training [19–21] or a lack of effective local preparedness and response [3, 18, 22, 23].

Beyond the lack of DRR implementation, there is also a lack of systematized DRR categorization [10, 24]. The following paragraphs explain two current DRR categorization systems and argue for a need for more systematized categorization of DRR strategies.

One current DRR categorization system divides disaster risk into its three components: hazard, exposure, and vulnerability. First, hazards are measured by their frequency and severity [3]. When the severity of the hazard cannot be reduced, limiting exposure and vulnerability to the hazard is key [3]. For instance, effective early warning systems and preparedness planning can reduce effects of hazards through actions such as timely evacuation [3]. Addressing exposure and vulnerability requires attention to the underlying causes of risks such as poverty, urban resiliency, environmental degradation, and inequality [3, 25]. Additionally, understanding how social and environmental determinants of health affect communities pre- and post-disaster is vital to creating “interventions [that] tackle systematically reproduced conditions of vulnerability” [26].

DRR can also be viewed from an additional categorization perspective, which isolates the three aspects of risk reduction: preventing future risk (prospective), reducing existing risk (corrective), or strengthening resilience to risk (compensatory) [9]. Both prospective and corrective DRR are necessary to reduce future disaster risk or to respond to current ones, but sometimes “technical protection measures are not fully reliable” [27] or corrective mechanisms cannot be immediate. In those instances, compensatory DRR aims to “strengthen the social and economic resilience of individuals and societies in the face of residual risk that cannot be effectively reduced” [9].

DRR strategies have not been adopted extensively and in instances where they have been, the strategies show a lack of coherence between the current categorization systems, despite that balanced and comprehensive DRR may lead to sustainable and effective interventions [10, 28].

In addition to incomplete adoption of DRR strategies, the terminology used to describe DRR has been inherently inconsistent. Creating effective DRR policies requires understanding disaster risk, which is currently hindered by “lack of standardized methodologies and definitions for the inclusion of disasters and [by lack of] robust impact measurement methodologies” [22]. This lack of standardized definitions also leads to challenges in recording and analyzing DRR, especially as it relates to health, whose indicators are complex and multifaceted [24]. Such analyses examining the strengths and weaknesses of current DRR mechanisms are necessary for the development of national DRR strategies [29]. An example of inconsistent terminology is the use of “prevention” as a blanket term for actions prior to a disaster [29, 30]. However, the term “prevention” could mean preventing the hazard from occurring, preventing people from being exposed, or preventing people from becoming vulnerable and unable to deal with exposure. The UN Technical Guidance Report on Monitoring and Reporting Progress of the Sendai Framework [31] does highlight the components of risk (hazard, exposure, vulnerability) and types of DRR strategies (prospective, corrective, compensatory). However, we observe that the document omits the intersection between these two categorization systems, despite the need for such an integrated framework. This need is evident in the Sendai Framework for DRR, which calls for “multi-hazard and multisectoral” preventative methods [8]. As such, we argue that this gap can be filled by creating and testing a novel framework that highlights the intersection between the three components of risk (hazard, exposure, vulnerability) and the three risk reduction approaches (prospective, corrective, compensatory). Creating a formal distinction between the different types of DRR is beneficial because it informs policy makers and other stakeholders of DRR gaps and duplicate interventions, allowing for more cost-effective planning. The aim of this current paper is to present and test a DRR categorization (referred to as “the proposed framework”) which could provide various stakeholders with information necessary to decide where to place limited resources and how to best plan for sustainable and effective DRR strategies. This proposed integrated framework could potentially be used to systematically evaluate DRR interventions for missing elements, leading to more holistic and balanced DRR approaches.

The novel integrated DRR framework conceptualized by the authors aims to aid in design of more comprehensive DRR policies. In order to reach this aim, the following research question was also addressed:

Does categorizing DRR interventions using a novel integrated framework provide new insights about the extent of DRR comprehensiveness?

## Methods

In order to answer the research question, a novel DRR framework was first conceptualized. Next, the proposed framework was tested by applying it to the 2019 Cities100 report. This study followed a mixed methods design and was conducted between January 2022 and May 2022.

### Study design and setting

The novel DRR framework was conceptualized following a grounded theory approach, which allowed for iterative and flexible qualitative data collection and analysis, fostering innovation throughout the process. Data collection at this phase stopped when the authors conceptualized an integrated DRR categorization.

Next, the novel integrated DRR framework was tested following a conceptual content analysis design. The transformative paradigm was used to answer the research question. Qualitative data was extracted from the 2019 Cities100 Report [32], whose data covers 58 cities spread globally. The report is released annually by C40 Cities Network, whose main funders are Bloomberg Philanthropies, Children’s Investment Fund Foundation, and Realdania, a non-profit philanthropic association. The document was chosen because it systematically describes DRR interventions while other reports focused on a single intervention and they did not associate interventions to their multiple effects, such as socioeconomic consequences. Without seeing the association between interventions and potential socioeconomic consequences, the analysis would yield significantly skewed conclusions about the use of the framework across a range of DRR categories. Additionally, the 2019 Cities100 Report has a more diverse international focus compared to other similar reports, which are not as representative of the global population. Ensuring geographic variance increased trustworthiness and internal and external validity through reduced selection bias.

In 2019, the Cities100 report identified ongoing interventions implemented by each city, the unit of study in our research. Out of the 58 cities in the report, the authors manually selected 25 cities for this particular research question. Criterion purposeful sampling was used to select all of the cities that implemented interventions relevant to air pollution. The criteria for selection were the words “air” or “pollution”, or the presence of air pollution as a downstream effect of the intervention. The presence of a single criterion qualified the intervention to be selected. For instance, divesting away from fossil fuels would decrease air pollution even if the phrase “air pollution” was not specifically used. The selected cities are located in 15 countries, seven geographical regions, and span four income-level classifications.

The specific makeup of the chosen data set was analyzed using descriptive statistics to give insight into the distribution across geographical regions and income levels. Additionally, further descriptive statistics were used to show the distribution of DRR interventions across the proposed framework. The analysis was conducted in Sweden using Excel [33] by Mariya Dimitrova. Megan Snair verified data quality using iterative proofreading to ensure that the interventions from the 2019 Cities100 Report were categorized properly. The study complied with the Standards for Reporting Qualitative Research (SRQR) guideline [34]. Establishing the novel integrated framework and its subcategories prior to data analysis was vital to ensuring data quality. Trustworthiness was enhanced through strengthened internal validity. For instance, the criteria within each DRR category, explained in the section "Creating a novel integrated framework to classifying DRR" , was clearly defined prior to data analysis. Additionally, pilot testing was conducted prior to data collection and analysis. Frequent monitoring of data collection and analysis by Megan Snair prevented duplicates, inconsistencies, analytical errors, and bias, including selection bias.

### Creating a novel integrated framework to classifying DRR

The novel integrated framework (Table 1) cross tabulates two current classification frameworks, whose intersections are populated with examples to illustrate each type of DRR strategy.

**Table 1:** The intersection between various components of disaster risk and types of DRR (Adapted from [3, 8])

**Novel Integrated DRR Framework**			
	**Prospective DRR**	**Corrective DRR**	**Compensatory DRR**
**Hazard** e.g., high air pollution	**prevent future hazard from occurring** e.g., investing in renewable energy sources to prevent future air pollution	**mitigate current hazard levels** e.g., reducing existing air pollution by replacing diesel vehicles with electric vehicles that are powered by renewable energy	**increase resilience to hazard (general population or community)** e.g., making polluted cities more livable, such as by increasing the number of green spaces to allow exposed residents to more easily cope with high air pollution
**Exposure** e.g., people live in a highly polluted city	**prevent future exposure** e.g., create support for people to not have to move into polluted cities, preventing overpopulation in an area exposed to air pollution	**mitigate current exposure** e.g., an intervention (such as making rural areas more attractive) that support people to move out of overpopulated and polluted cities; e.g., transfer the hazard itself (such as a polluting factory) away from a densely populated area to a less populated area	**increase resilience to past or present exposure (general population or community)** e.g., preparedness actions, such as a warning system plan for days with very high air pollution
**Vulnerability** e.g., people who are impoverished or have a respiratory illness are unable to properly cope with the effects of air pollution	**prevent future vulnerability from occurring (focus on specific vulnerable groups)** e.g., vulnerability prevention within low-income areas: policy change to prevent deeper inequalities	**mitigate current vulnerability (focus on specific vulnerable groups)** e.g., policies that mitigate inequality and power imbalances. These interventions attempt to correct present vulnerabilities	**increase the resilience of those who are particularly vulnerable (focus on specific vulnerable groups)** e.g., financial assistance to single mothers who live in highly polluted cities; e.g., communicate with high-risk individuals (people with asthma or chronic respiratory diseases) to stay at home during days with critical levels of air pollution

In the first row, *“Hazard, prospective”* categories aim to prevent the hazard from occurring. *“Hazard, corrective”* categories attempt to mitigate current hazard risk. *“Hazard, compensatory”* interventions strive to increase the resilience solely to hazards. As shown in the examples, while all interventions seek to reduce the hazard of air pollution, the prospective approach is a broad investment across renewable energy, the mitigation focus is to specifically remove a known form of air pollution (diesel vehicles) and replace with a less pollutive option (electric vehicles), and the compensatory action is to increase resilience to the hazard of pollution by increasing green spaces.

In the second row, a DRR strategy that targets exposure aims to decrease how much or how often people or assets are in the way of hazards. Such interventions pre-emptively separate the hazard from people or assets, but they do not remove the hazard altogether. Moving across the row, *“Exposure, prospective”* occurs when an intervention attempts to prevent broad future exposure. *“Exposure, corrective”* categories attempt to reduce current exposure, as demonstrated in the example of moving current residents from the city out to more rural areas or removing the source of pollution, where it is possible to “correct” the exposure. *“Exposure, compensatory”* increase resilience to exposure.

Finally, the third row focuses on DRR strategies that target vulnerability of individuals or communities that are particularly susceptible to the effects of a disaster. Moving across the row, *“Vulnerability, prospective”* attempts to prevent the vulnerability from occurring. Conversely, *“Vulnerability, corrective”* categories mitigate *current* vulnerability. This category differs from the “exposure, compensatory” category, which focuses on the general population rather than a select few vulnerable individuals or groups. Finally, *“Vulnerability, compensatory”* categories might be necessary when the vulnerability itself cannot be mitigated. Thus, these interventions attempt to increase the resilience of people who are particularly vulnerable, compensating for current vulnerabilities whose root causes might not be possible to address immediately.

### Testing the novel integrated approach to classifying DRR

First, data was extracted from the 2019 Cities100 report by creating a list of interventions for each city. New York City (NYC), for instance, lowered the speed limit and expanded bike lanes in 2013. These programs could be counted as one intervention (encourage residents to bike) or they can be counted as two distinct interventions (lowered the speed limit and expanded bike lanes). Because the report described these as two separate initiatives, the analysis also counted them as two interventions.

Next, deductive conceptual content analysis was used to place/connect each public health intervention into the DRR categories of the proposed DRR framework from Table 1. The term “connection” indicates that an intervention can be categorized into a particular section of the novel integrated approach. The term “connection” was chosen to highlight that this is not a mutually exclusive method: a single intervention could fit into multiple DRR categories, so there could be several connections. Including only the explicitly stated health connections was deliberate as it implied that the authors of the Cities100 report had considered only those to be present. However, our content analysis did not search for specific words or phrases, but rather focused on the presence of concepts, which is why our content analysis is conceptual. This analysis aided in understanding the extent to which the Cities100 Report successfully used DRR explanations as motivations for their interventions. Each connection was given a value of one for the continuous data set. Examples from this list of interventions is shown in the Appendix.

A dichotomous data set was also created, where each city, rather than intervention, received a value of one if the city used a DRR category at least once. This was done to account for variations in how interventions were split up and counted, as in the example from NYC above.

Furthermore, the dichotomous data set was analyzed using the following equation:

1 $$\mathrm{pDRR }= \left(\frac{number\, of\, DRR\, connections }{number\, of\, maximum\, potential\, DRR\, connections} \,x\, 100 \right).$$

where “pDRR” stands for a percentage of city-achieved DRR connections. The number of maximum potential DRR connections for each intervention is nine: each intervention can potentially fit into all nine categories of the proposed DRR framework. Therefore, the total number of maximum potential DRR connections is the number of interventions times nine. The resulting percentage accounts for the over-representations of certain regions and income levels because it considers the total number of possible DRR connections from the proposed DRR framework, and not just the sum number of connections presented in the 2019 Cities100 Report. Excel was used to apply this formula, calculating the distribution of city-achieved DRR interventions for two variables: region and FY23 World Bank country income-level [35].

## Results

**Fig. 1:**
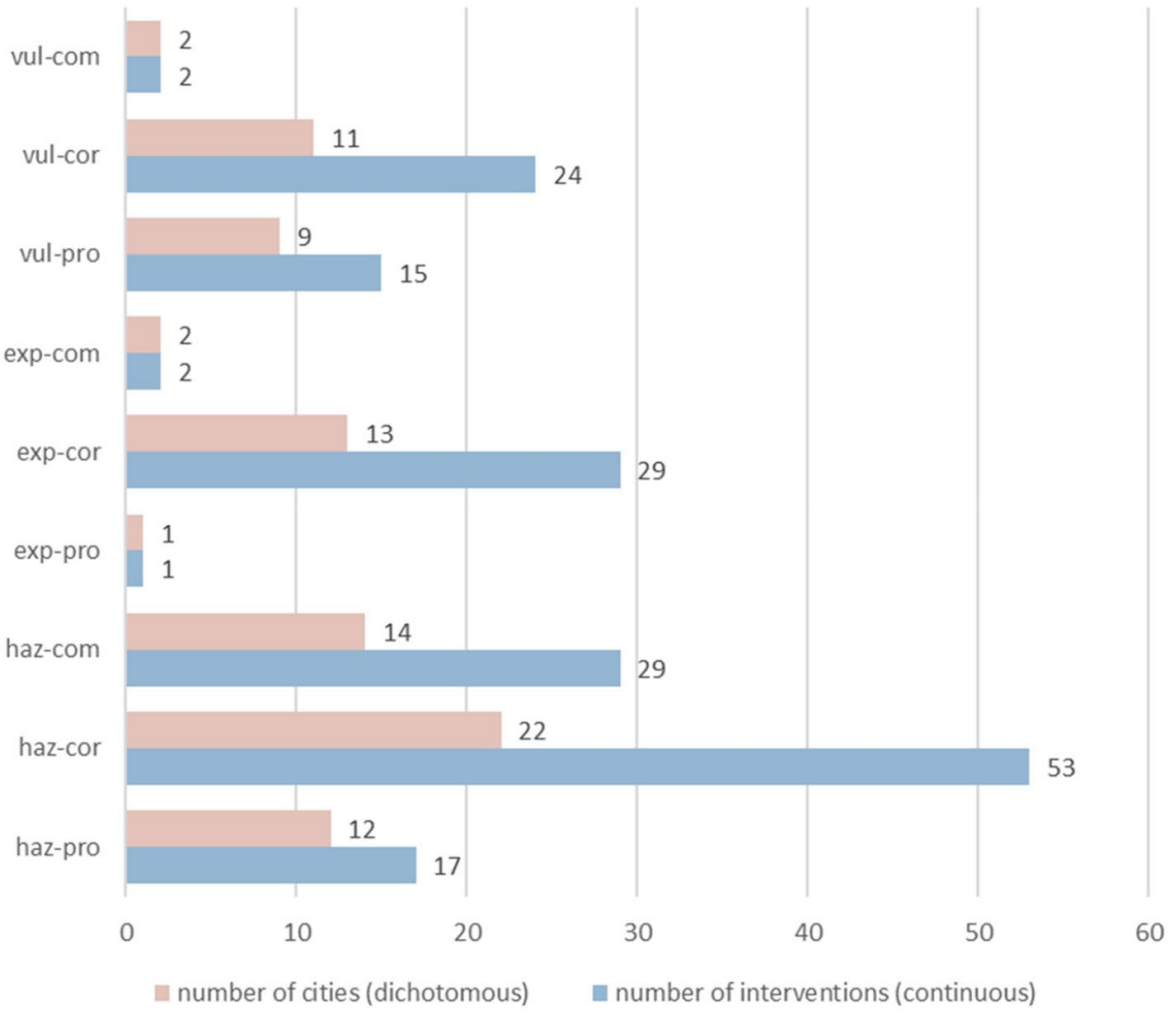
Total number of cities and interventions utilizing each DRR strategy

Out of all nine DRR categories presented in the integrated DRR framework (Table 1), the “exposure, prospective” category was the least implemented/explained, followed by the “vulnerability, compensatory” and “exposure, compensatory” categories (Fig. 1). In contrast, the “hazard, corrective” category was the most commonly used category by the cities analyzed from the 2019 Cities100 Report (Fig. 1).

**Fig. 2:**
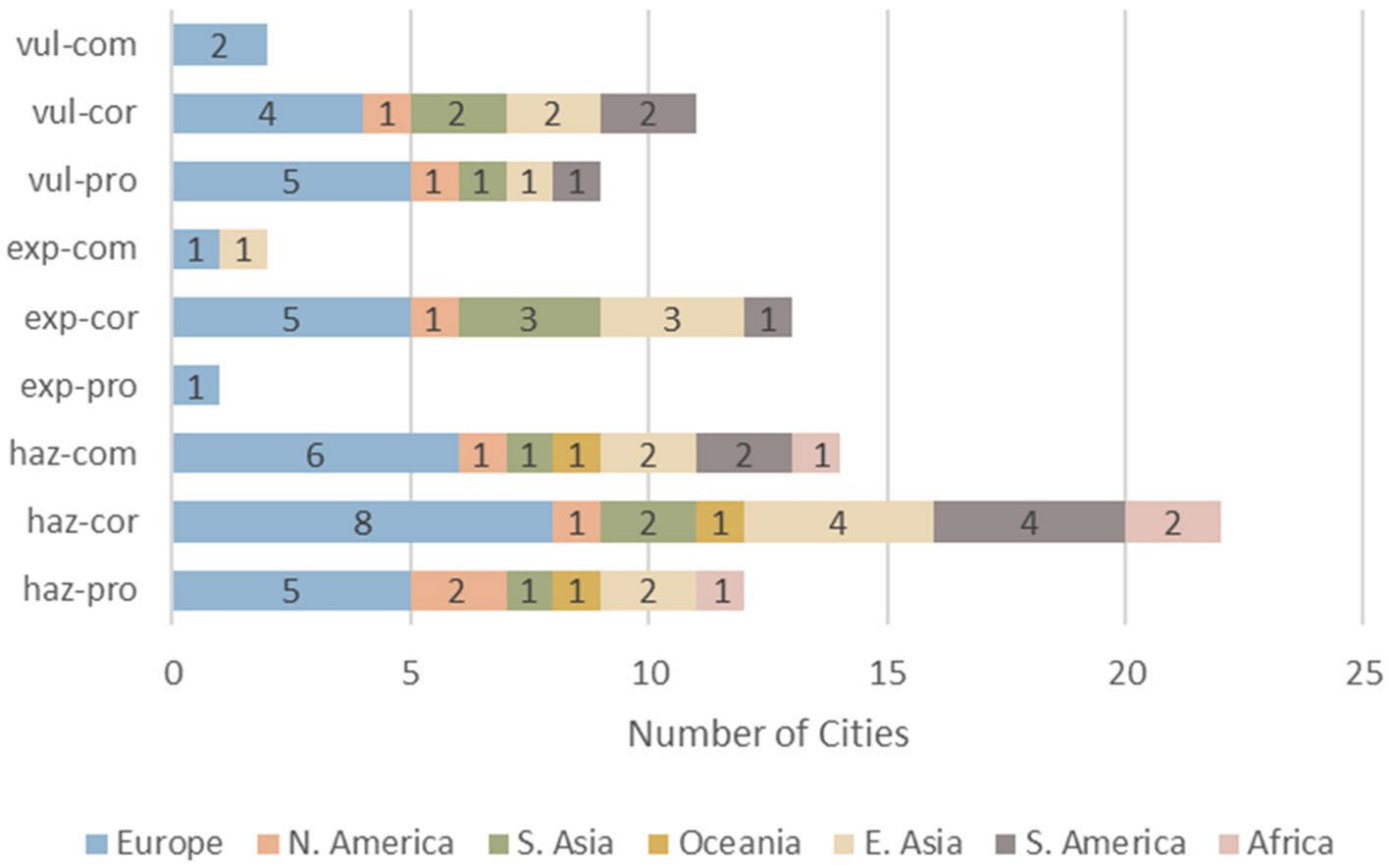
Number of cities, grouped by region, implementing each DRR strategy

**Fig. 3:**
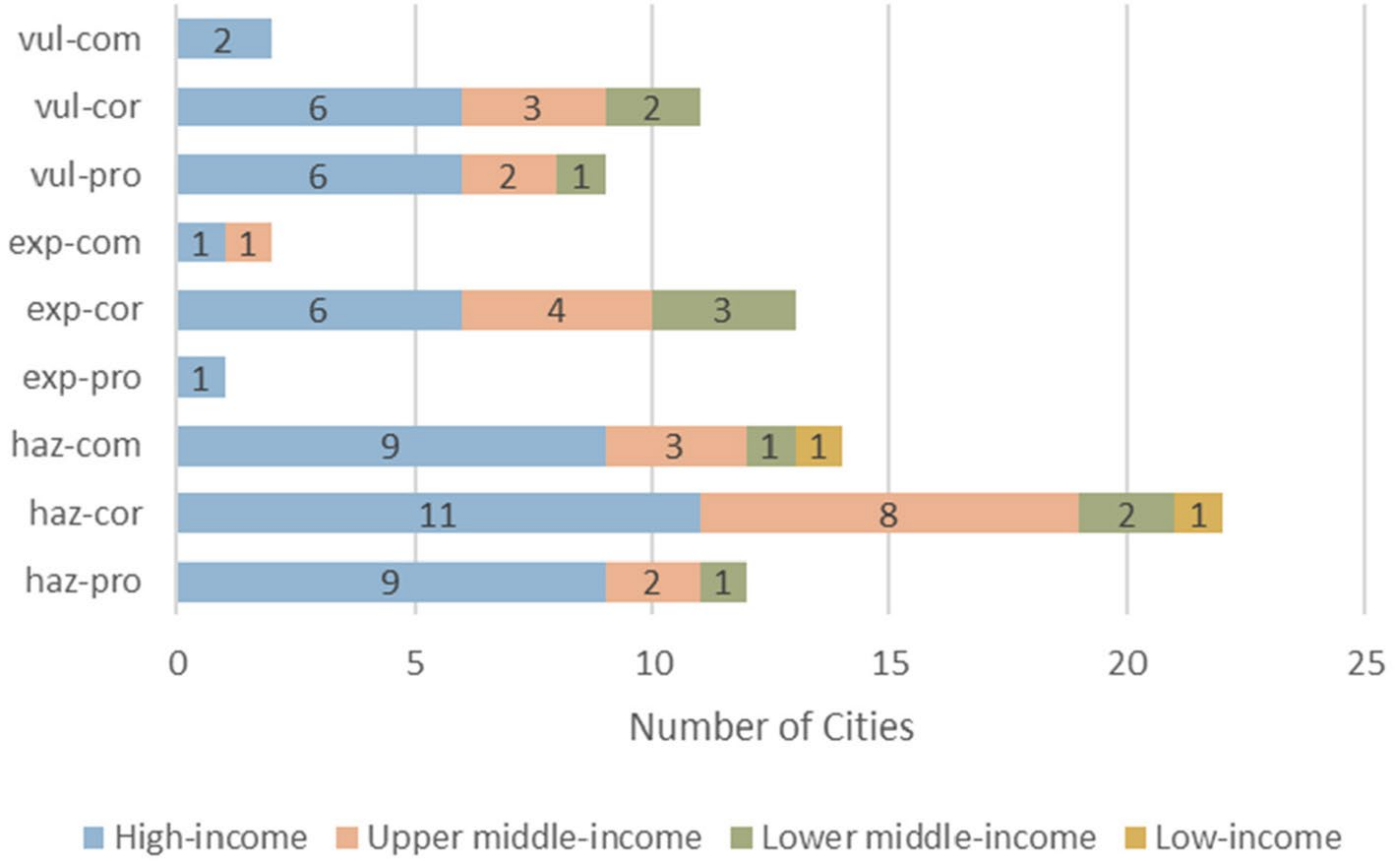
Number of cities, by country income level, implementing each DRR strategy

**Fig. 4:**
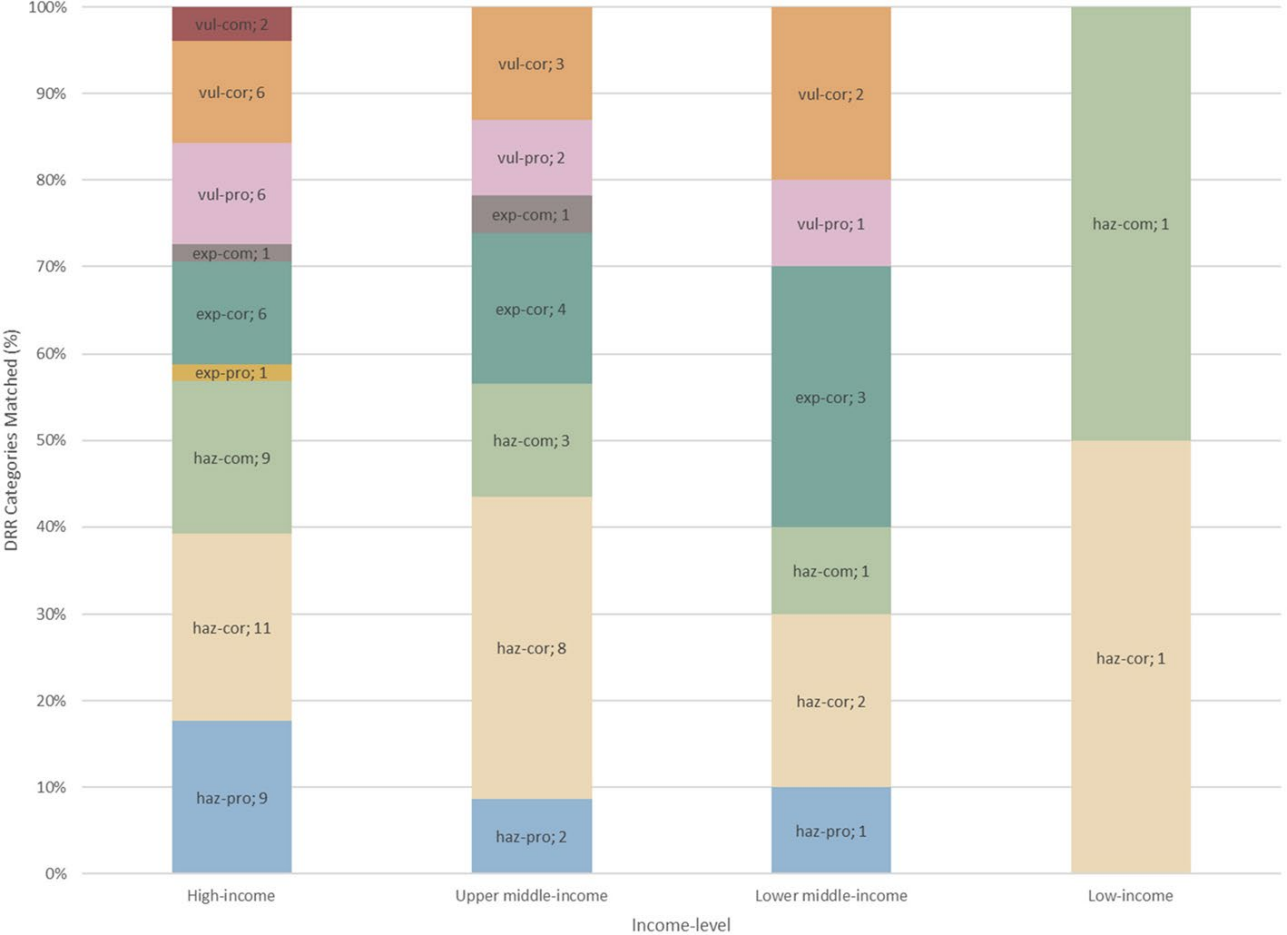
DRR category makeup of each income-level group

Similarly, Figs. 2 and 3 also confirmed the trends seen in Fig. 1. European, high-income, and upper middle-income cities contributed the most to every DRR category compared to the other analyzed regions (Figs. 2 and 3). Upper middle-income countries favored “hazard, corrective” strategies over other DRR categories while lower middle-income countries favored “exposure, corrective” over other DRR strategies (Fig. 4).

**Fig. 5:**
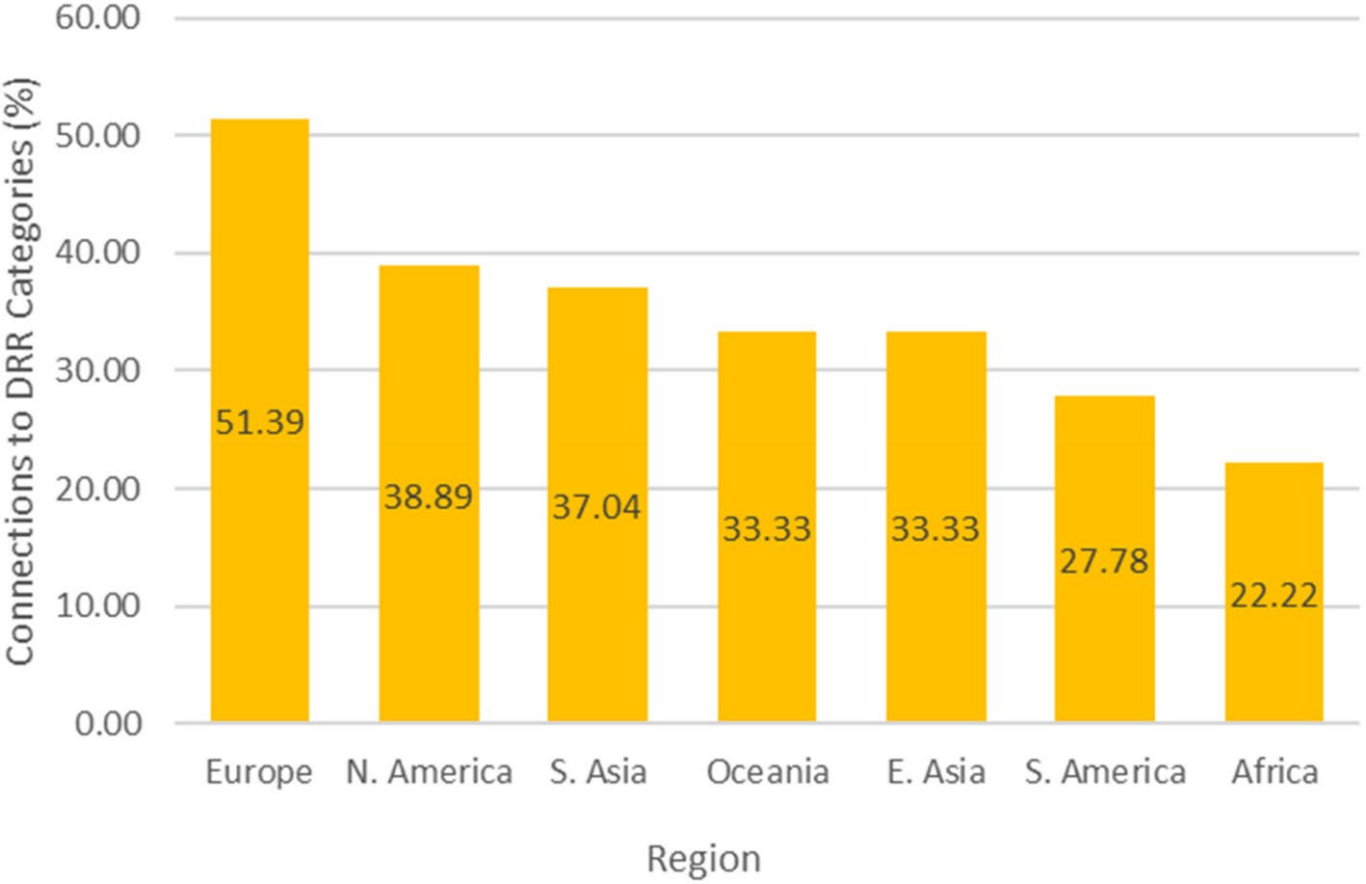
Percentage of City-Achieved DRR Connections, Grouped by Region

**Fig. 6:**
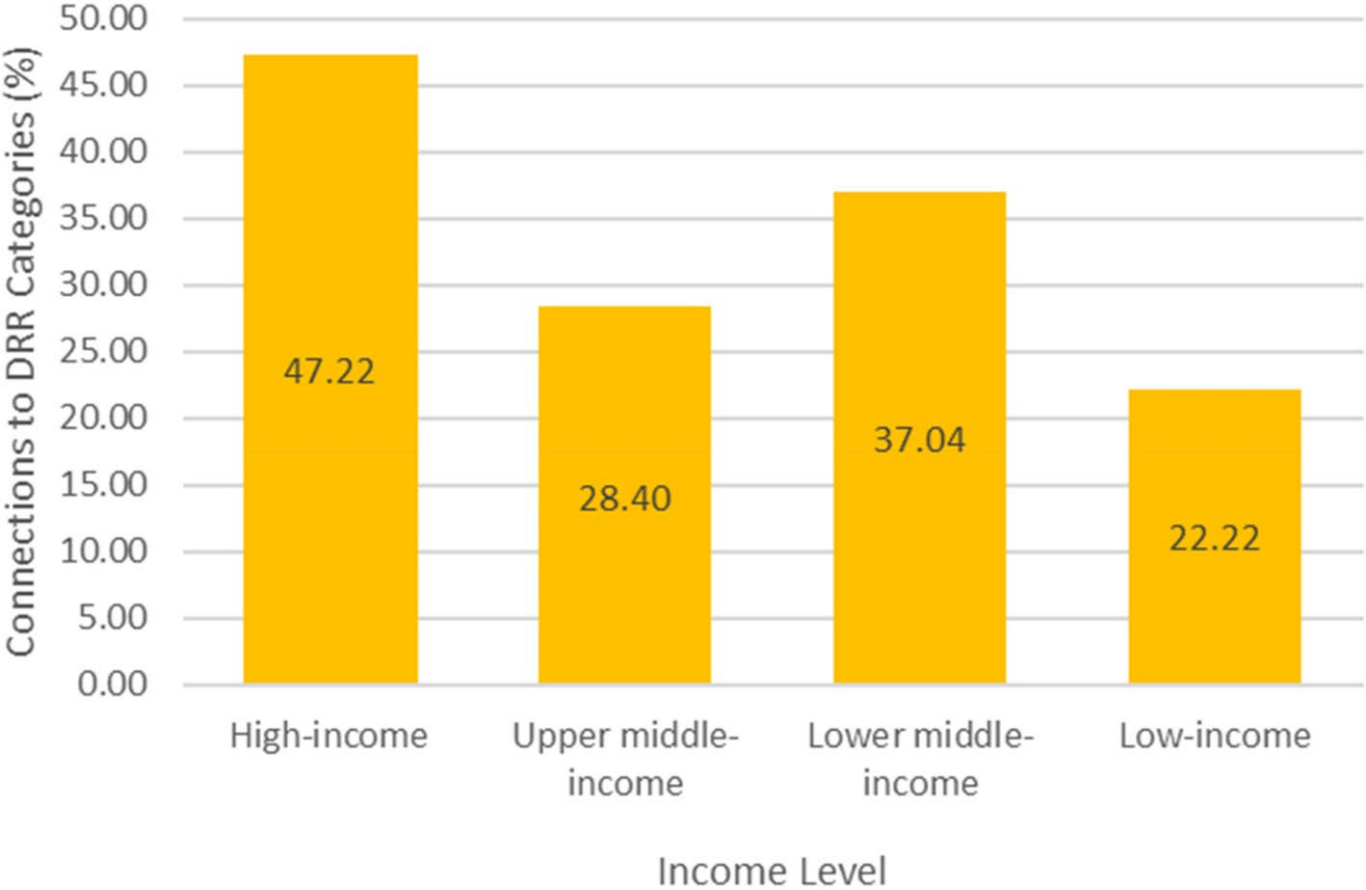
Percentage of City-Achieved DRR Connections, Grouped by Income-Level

**Fig. 7:**
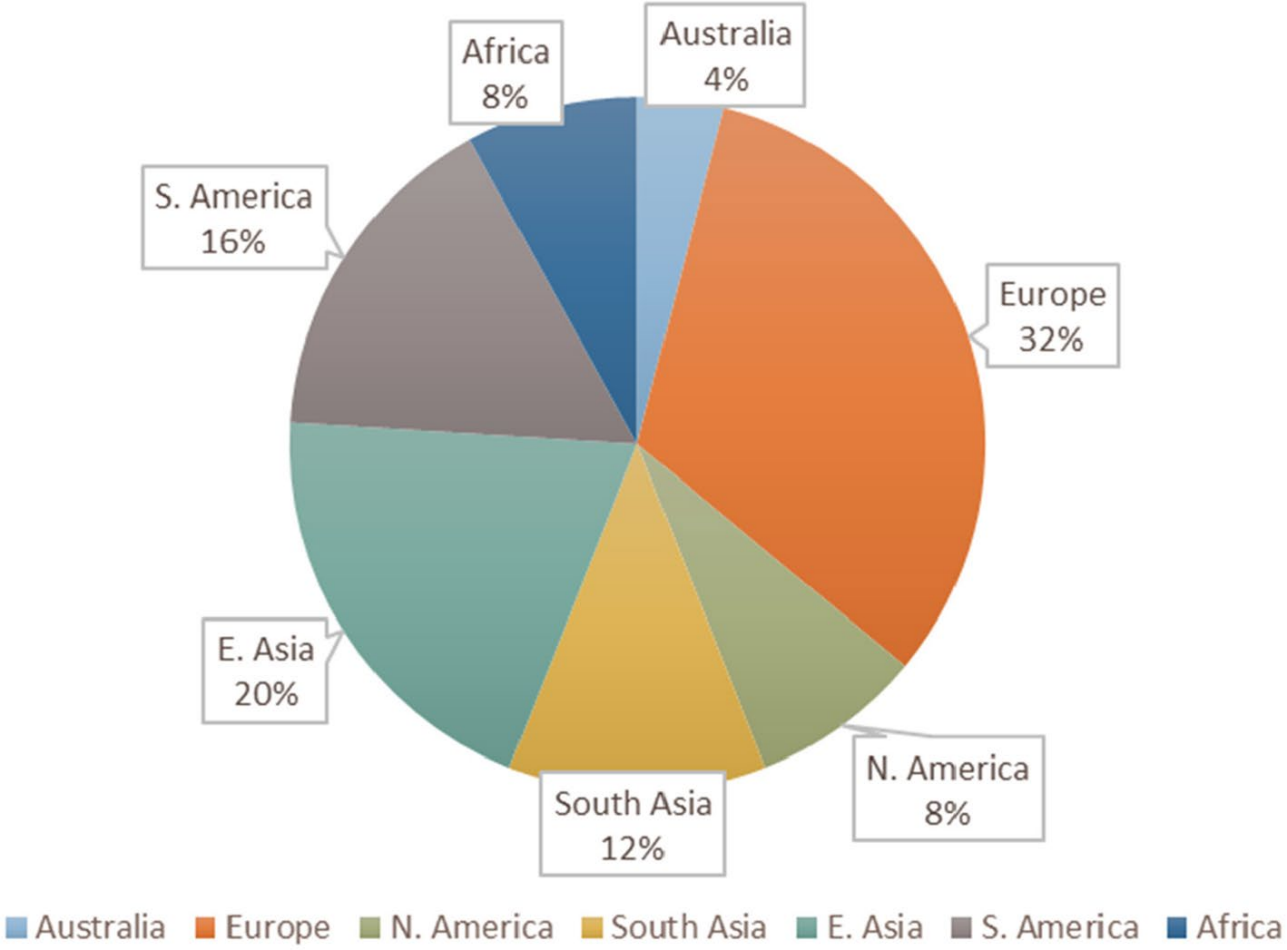
Percentage of regional representation within the analyzed data set

**Fig. 8:**
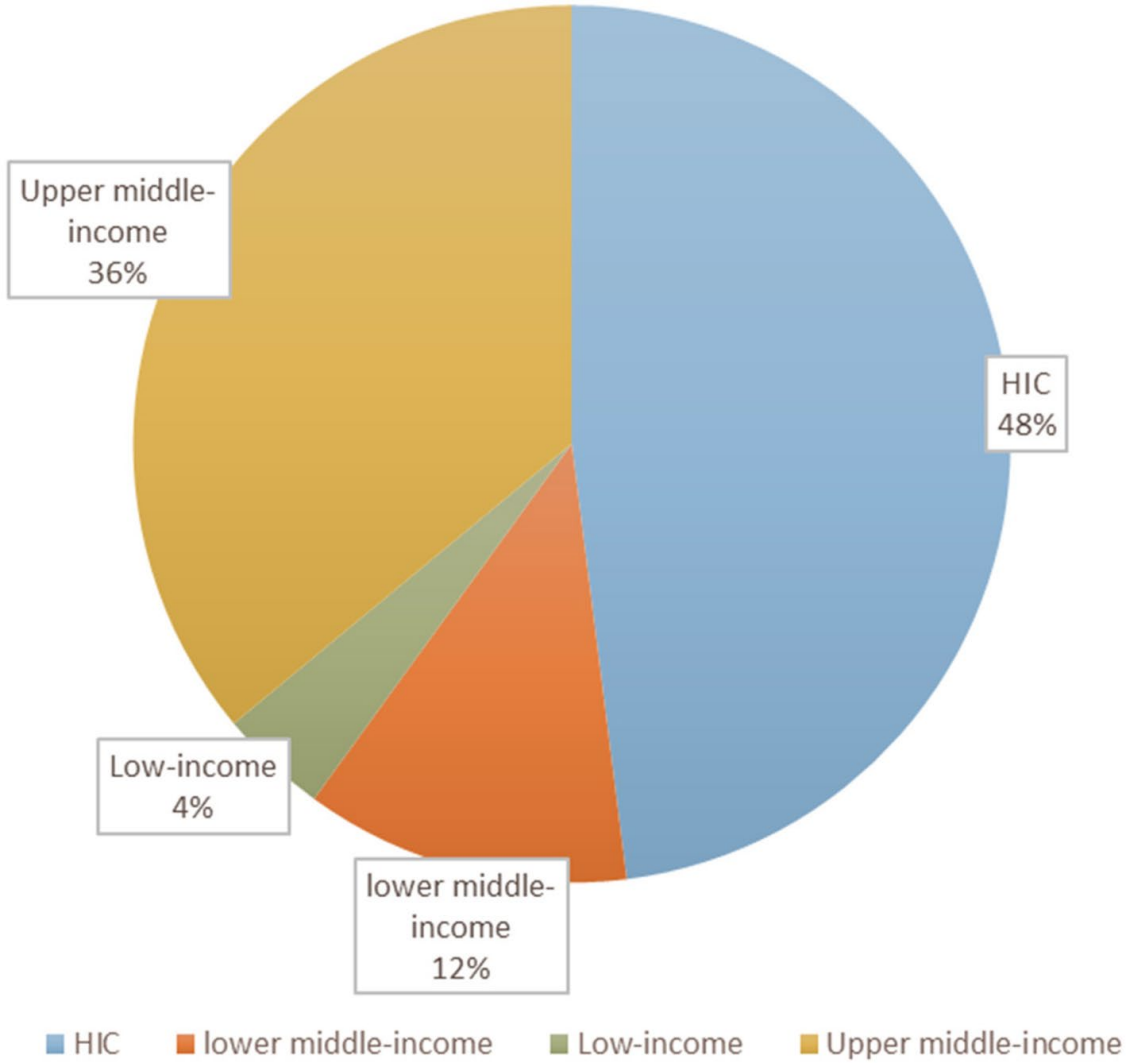
Percentage of income-level representation within the analyzed data set

Figure 5 also indicates that European cities had the highest percentage of DRR connections (51.39%) compared to the maximum possible DRR connections, while African cities had the lowest percentage of DRR connections (22.22%). Cities in high-income countries had the largest percentage of DRR connections: they implemented 47.22% of all available DRR categories, followed by cities in lower middle-income countries (37.04%), upper middle-income countries (28.40%), and finally low-income countries (22.22%) (Fig. 6). Overall, European countries accounted for 48% of the data points analyzed (Fig. 7) and high-income countries accounted for 48% (Fig. 8).

## Discussion

Analysis of the 2019 Cities100 Report using this novel approach exposed gaps within the selected interventions: several DRR categories were under-utilized, and some regions were able to integrate more DRR categories compared to other regions. The current analysis provides important insight into DRR strategy gaps, indicating that the novel integrated framework can potentially serve as a useful analytical tool.

### Trends from the analysis using the novel integrated DRR framework

Within the 2019 Cities100 Report, one important trend observed was that the “hazard, corrective” category was most frequently used, especially by upper middle-income countries, compared to the other categories (Figs. 1, 2, 3 and 4). A reason for this might be that “hazard, corrective” interventions were cheaper and politically easier to achieve [13] because they place the burden on individuals rather than systemic issues. For instance, numerous countries encouraged residents to bike or recycle as a means of mitigating inner-city pollution (Appendix). Nevertheless, interventions that targeted systemic or organizational issues were in fact sometimes present within the “hazard, corrective” category. For instance, Delhi shut down the most polluting inner-city power stations and Stockholm recycled heat from data centers and used it for heating homes. However, these programs fail to remove dependency on polluting sources of energy because they relocate the pollution rather than removing its presence or preventing its creation, which would require “hazard, prospective” interventions. Doing so would require investment in renewable energy, an intervention that belongs to the “hazard, prospective” category. The novel DRR framework could be a useful analytical tool that uncovers a deficit of specific DRR categories, such as “hazard, prospective”, within the 2019 Cities100 Report.

Another trend observed was that the “exposure, corrective” category was significantly more represented than the other two exposure DRR categories (Fig. 1). This finding is in line with previous research, which points to “challenges of implementation and a lack of investment in preventive action” [36]. The term “preventive” as used by previous research can fit into the “prospective” or “compensatory” categories within the proposed DRR framework. Therefore, it is logical that corrective actions would be overrepresented, as seen in Fig. 1. One reason for the observed trend might be that addressing resilience to potential future exposures (“exposure, prospective” category) requires more planning and political will than reacting to current threats [16]. Additionally, addressing vulnerabilities that cannot be immediately mitigated (”exposure, compensatory” category) through resilience-building requires significant financial investment and the resulting change takes years to realize, making it difficult to justify such funding, especially in the face of immediate threats [19]. As discussed in the introduction, both of these actions are politically unappealing and it would be difficult to mobilize support for them. Moreover, focusing limited financial resources on vulnerable populations could be seen as unfair or wasteful, while resilience-building of the general population might be favored [19]. Continued public education and awareness campaigns on the importance of diverse DRR interventions can help build support for more balanced investments.

Finally, the novel integrated DRR framework revealed regional trends. European cities constituted the largest percentage of overall DRR connections (Fig. 5). Lower middle-income countries had higher percentage of DRR connections compared to upper middle-income countries, despite being in a lower income bracket (Fig. 6). Additionally, lower middle-income countries favored “exposure, corrective” DRR categories over other DRR categories, possibly because financial resources and political coordination are especially limited in lower middle-income countries, leaving mitigation of the exposure as a more viable option [37].

### Strengths and weaknesses of the study

A limitation of the current analysis, which was done using the 2019 Cities100 Report, is that each description of a city intervention takes up exactly two pages within the original 2019 Cities100 report, meaning there was limited information for the authors to extract. Perhaps a longer explanation of each city plan might have contained more relevant information about the interventions or health outcomes, allowing for the authors of this study to deduce more DRR connections. In fact, some interventions utilized DRR categories that were not acknowledged in the explanation. For instance, many of the interventions listed financial gain as a potential benefit, but there was no explanation as to how this financial gain could lead to greater resilience. For instance, Delhi, India provided subsidies for rickshaw drivers to purchase electric rickshaws, mitigating overall city pollution as well as limiting driver exposure and proximity to tailpipe air pollution. However, the writers of the 2019 Cities100 Report did not mention that subsidizing impoverished rickshaw drivers could potentially increase their resilience in the face of an already-polluted city and prevent further vulnerability. Because the link was not explicitly made, the intervention was not counted in our analysis as “vulnerability, corrective” or “vulnerability, prospective.” If such connections were made in the description, or longer timelines were allowed between measurement of intervention and effect, different rate and distribution of DRR utilization may have been observed. Alternatively, the authors of this current paper could have utilized external sources to obtain elements missing from the 2019 Cities100 Report. However, that would alter the results of the DRR analysis because we would introduce data points from outside of the 2019 Cities100 Report. The aim of this current paper was to test the ability of the proposed DRR framework to analyze a single DRR strategy or report, rather than a compilation of DRR reports. This limitation of the 2019 Cities100 Report, however, does not impact the creation, future utilization, or reproducibility of the proposed DRR framework because application of the approach to a different report would offer a different level of detail.

A barrier to implementing the proposed DRR framework is that it requires additional time and resources that might not always be available. First, developing the necessary data sharing and coordination mechanisms needed to compile DRR interventions in a jurisdiction can be difficult in the face of organizational silos. Second, the analysis can be time- and resource-intensive because if the hazard changes, the corresponding actions and potential interventions throughout the framework would change as well. Third, underutilization of a certain DRR category does not necessarily mean that it should be implemented immediately. For instance, encouraging people to bike in heavily polluted cities exposes bikers to higher levels of air pollution, which creates a further vulnerability, so careful examination of consequences to implementation is needed. Finally, the benefit–cost ratio of interventions could vary depending on context [38]. Therefore, contextual analyses might be useful in evaluating whether adding a certain DRR category would be beneficial in certain environments.

### Future research

Future analysis of a larger or more diverse data set or a variety of hazards could improve statistical significance, increasing the potential generalizability and external validity of the observed trends. Alternatively, application of the proposed DRR framework to a more thorough report or usage of multiple sources could also provide a different level of detail. Additionally, future research could investigate which barriers, such as political feasibility, lack of knowledge, high cost, or slow return on investments, should be addressed to encourage uptake of under-utilized DRR categories. Future focus on low- and middle-income countries might reveal unique gaps in those regions, as this analysis was overly focused on European and high and upper-middle income cities. This information might be particularly useful because limited resources require efficient policy planning to best reduce disaster risk, especially in areas more prone to the effects of climate change or navigating the risks within a fragile state [39]. Finally, it might be useful to analyze the same interventions from various angles/hazards in order to gain full perspective of missing DRR elements.

### Conclusions and implications

The current analysis indicates that the proposed DRR framework is useful in categorizing DRR interventions and in elucidating which DRR strategies are under- and over-utilized. For instance, this analysis points to a need for increased and more diverse implementation and/or explanation of DRR categories within African and South American cities (Fig. 5). Additionally, the analysis indicates an over-utilization of “hazard, corrective” DRR categories (Fig. 3), especially in upper middle-income countries (Fig. 4) compared to other DRR categories, as well as an over-utilization of “exposure, compensatory” DRR categories compared to the other compensatory categories, which would target hazard and vulnerability (Fig. 2). The analysis suggests the “hazard, compensatory,” “exposure, prospective,” and “vulnerability, compensatory” categories were under-utilized in this analysis, relative to the other categories from the novel DRR framework (Fig. 2). Further research is necessary to understand whether including such DRR categories would be beneficial to a specific context. Nevertheless, current literature indicates that comprehensive DRR strategies are more sustainable and effective and more balanced implementation may result in better outcomes [16, 24, 26, 30]. Application of the proposed DRR framework elucidates trends and gaps present in the 2019 Cities100 Report, provides insight into where future efforts should focus, and indicates that an integrated framework might be useful in creating more equitable and sustainable DRR.

## Data Availability

All data generated or analysed during this study are included in this published article [and its supplementary information files].

## Appendix

### Examples of DRR interventions from the 2019 Cities100 Report

A non-exhaustive list of intervention examples from the analysis of the 2019 Cities100 Report using the integrated DRR framework. The city, country, and start year of the intervention are listed. The start year of the intervention is taken from the 2019 Cities100 Report, unless there is a footnote. For interventions with a footnote, the 2019 Cities100 Report did not include a start year and we used external sources to obtain the start year.

**Table:**

	**Prospective** DRR activities are those “that address and seek to avoid the development of new or increased disaster risks.”	**Corrective DRR** “activities [are those] that address and seek to remove or reduce existing disaster risks.”	**Compensatory** “activities [are those that] strengthen the social and economic resilience of individuals and societies in the face of residual risk that cannot be effectively reduced.”
**Hazard** is “a process, phenomenon or human activity that may cause loss of life, injury or other health impacts, property damage, social and economic disruption or environmental degradation.” e.g., high air pollution	**prevent future hazard from occurring** e.g., provide docked ships with renewable energy so they do not run their engines. This invests money in renewable energy, while avoiding air pollution of the city (Stockholm, Sweden - 2019^a^) e.g., require large municipal buildings to install solar panels, assist poor households in purchasing solar panels and cheaper renewable energy (Delhi, India - 2013^b^) e.g., divest pension funds from fossil fuels (NYC, USA - 2019) e.g., use seawater to cool buildings. This removes the need for traditional cooling units, which require much more electricity and emit more CO^2^ (Hong Kong, China - 2019) e.g., encourage use of low-carbon concrete (Honolulu, USA - 2019^c^) e.g., establish research hub for renewable energy (Copenhagen, Denmark - 2015^d^)	**reduce current hazard levels** e.g., reduce the number of total vehicles (Stockholm, Sweden - 2014) e.g., increase the number of people biking, walking, using public transportation using various methods, such as making streets safer or making bike sharing accessible and affordable. Increased biking, walking, use of public transportations decreases the number of diesel vehicles (NYC, USA - 2013), (Guangzhou, China - 2018), (Copenhagen, Denmark - 2012), (Bucaramanga, Colombia - 2016), (Bogota, Colombia - 2016), (Bengaluru, India - 2013), (London, UK - 2018), (Bologna, Italy - 2018^e^), (Addis Ababa, Ethiopia - 2018^f^), (Fortaleza, Brazil - 2013) e.g., Fine idling vehicles who run their engines longer than 3 min by the curb and 1 min in a school zone (NYC, USA - 2017) e.g., shut down the dirtiest power stations and decrease use of coal (Delhi, India - 2018^g^), (Chengdu, China - 2013) e.g., reduce dust from inner-city construction (Chengdu, China - 2013) e.g., educate city residents about the effects of air pollution and about what they can do to reduce air pollution (Bologna, Italy - 2018^h^), (Milan, Italy - 2018^i^) e.g., create local programs that increase recycling and composting of materials, which reduces transportation emissions and avoids creation of new materials - Sydney (Australia), Sao Paulo (Brazil), (Paris, France - no data), (Naestved, Denmark - 2018^j^) e.g., transform a landfill into a forest, which sequesters CO^2^ emissions (Durban, South Africa - 2008^k^) e.g., retrofit buildings to be more energy efficient (NYC, USA - 2019), (Paris, France - 2015^l^) e.g., recycle heat from data centers and use it for heating of city homes - (Stockholm, Sweden - 2017^m^)	**increase resilience to hazard (general population or community)** e.g., encourage all people to bike more improves their cardiovascular health and allows them to better cope with current exposure to air pollution (Bogota, Colombia - 2016), (Copenhagen, Denmark - 2012) e.g., The space created by the absence of cars (due to low-emission zone) can be allocated for public use to create “a more enjoyable and livable city (Milan, Italy - 2019), (NYC, USA - 2013) e.g., creating car-free days once per month encourages residents to gather on the streets, building social cohesion (Addis Ababa, Ethiopia - 2018^n^) e.g., using seawater to cool building frees up rooftop space for roof top gardens, making the city more livable and spacious (Hong Kong, China - 2019^o^) e.g., improve bus stops to encourage small and medium businesses to the area, which is not necessarily vulnerable (Guangzhou, China - 2018)
**Exposure** “refers to the situation of people, infrastructure, housing, production capacities and other tangible human assets located in hazard-prone areas” e.g., people live where air pollution is high	**prevent future exposure** e.g., ensure that retrofitted homes can be affordable so as to not push out low-income residents into polluted areas (London, UK - 2018)	**reduce current exposure** e.g., replace diesel vehicles with electric ones that are not necessarily powered by renewable energy (Stockholm, Sweden - 2014), (NYC, USA - 2013), (Kolkata, India - 2017), (Guangzhou, China - 2018), (London, UK - 2019) e.g., introduce the “common mobility card” to make transfers easier between different modes of public transport and to lower “direct exposure to particulate matter” by decreasing the amount of time residents have to spend commuting (Kolkata, India - 2017) e.g., creating bus lanes, bus bays and nicer bus stops lower exposure to local air pollution due to lower commuter times (Bengaluru, India - 2013) e.g., provide docked ships with renewable energy so they do not run their engines. This invests money in renewable energy, while avoiding air pollution of the city (Stockholm, Sweden - 2019^p^) e.g., Fine idling vehicles who run their engines longer than 3 min by the curb and 1 min in a school zone (NYC, USA - 2017) e.g., create a low-emission zone in city centers to ban diesel and large vehicles. This decreases inner-city pollution and inner-city congestion, both of which expose city residents to polluted air (Milan, Italy - 2019), (London, UK - 2018) e.g., turn waste leachate into groundwater, preventing harmful emissions to be released into the environment (Bengaluru, India - 2016^q^) e.g., cooling buildings using seawater reduces local air pollution and reduces exposure to air pollution (Hong Kong, China - 2019) e.g., retrofit buildings using carbon-cutting measures. This reduces indoor exposure to air pollution (NYC, USA - 2019), (Paris, France - 2015).	**increase resilience to exposure (general population or community)** e.g., create an “emergency action plan for particularly bad air pollution events”, including a warning system for such days, and identify heavily-polluting industries that can be managed during such days. This intervention allows residents to cope with air pollution more effectively (Chengdu,China - 2013), (Bologna, Italy - 2018^r^)
**Vulnerability** includes “things that increase susceptibility to a disaster” e.g., people who are impoverished or have prior health conditions are unable to properly deal with the effects of isolation e.g., people who are poor or have prior health conditions are unable to deal with the effects of high air pollution or isolation if they live in those areas	**prevent *****future***** vulnerability from occurring (focus on specific vulnerable groups)** e.g., encouraging people to bike more improves their cardiovascular health and prevents future vulnerabilities to air pollution - (Bogota, Colombia - 2016), (Copenhagen, Denmark - 2012) e.g., Create retrofitting jobs that are accessible to low-income residents (NYC, USA - 2019) e.g., provide assistance to hospitals and low-income buildings to be retrofitted, becoming more energy-compliant. This intervention allows hospitals and low-income housing to comply without being overburdened, which might lead to further vulnerabilities (NYC, USA - 2019)	**reduce *****current***** vulnerability (focus on specific vulnerable groups)** e.g., create strict “ultra-low emission zone” to protect the most vulnerable residents, who experience higher amounts of air pollution but are less likely to own a car (London, UK - 2019) e.g., provide financial assistance to poor city residents, who often live in “energy poverty”, for the installation of solar panels. This intervention provides cheap and clean energy to those most in need, allowing them to cope with the negative effects of a polluted city (Delhi, India - 2016^s^)	**increase the resilience of those who are particularly vulnerable (focus on specific vulnerable groups)** e.g., Establish a fund to help “small- and medium-sized enterprises purchase less-polluting vehicles”, which helps those people who are not able to comply with inner-city clean vehicle rules (Milan, Italy - 2019)

^a^https://www.shippax.com/en/press-releases/the-power-is-on-at-the-vartahamnen-port-and-several-vessels-are-already-connected.aspx

^b^https://bridgetoindia.com/backend/wp-content/uploads/2019/02/BRIDGE_TO_INDIA_Rooftop_Revolution_Delhi_long.pdf

^c^https://www.reuters.com/article/idUSKCN1TX1K8/

^d^https://www.energylabnordhavn.com/

^e^https://www.fondazioneinnovazioneurbana.it/laboratorioaria/

^f^https://africa.itdp.org/ethiopia-marks-eleventh-car-free-day/

^g^https://timesofindia.indiatimes.com/city/delhi/badarpur-thermal-plant-shut-for-good/articleshow/66228551.cms

^h^https://www.fondazioneinnovazioneurbana.it/laboratorioaria/

^i^https://sdg.iisd.org/news/23-cities-and-regions-commit-to-pathway-towards-zero-waste/

^j^https://goexplorer.org/business-and-climate-initiative-for-entrepreneurship/

^k^https://unfccc.int/files/secretariat/momentum_for_change/application/pdf/1_buffelsdraai_reforestation.pdf

^l^https://vb.nweurope.eu/media/8346/ace_fiche_municipalities_paris_october2019_vf-finale.pdf

^m^https://theindexproject.org/award/nominees/1854

^n^https://africa.itdp.org/ethiopia-marks-eleventh-car-free-day/

^o^https://www.emsd.gov.hk/en/energy_efficiency/district_cooling_system/introduction/index.html

^p^https://www.shippax.com/en/press-releases/the-power-is-on-at-the-vartahamnen-port-and-several-vessels-are-already-connected.aspx

^q^https://timesofindia.indiatimes.com/city/bengaluru/bluru-scientist-converting-omans-black-water-into-white/articleshow/56226394.cms

^r^https://www.fondazioneinnovazioneurbana.it/45-uncategorised/1856-che-aria-e-monitora-e-migliora-la-qualita-dell-aria-di-bologna

^s^https://www.pv-magazine-india.com/2018/10/01/delhi-cabinet-approves-rooftop-solar-scheme/

## Abbreviations

DRR: Disaster Risk Reduction
NYC: New York City
SRQR: Standards for Reporting Qualitative Research
UN: United Nations
